# Implementing the Latvian Early Intervention Program (LAT-EIP) for Patients With Schizophrenia Spectrum First-Episode Psychosis: Study Protocol

**DOI:** 10.3389/fpsyt.2019.00829

**Published:** 2019-11-13

**Authors:** Liene Berze, Sandra Civcisa, Ilona Krone, Dmitrijs Kvartalovs, Sarmite Kikuste, Inna Sapele, Jelena Lazovika, Elmars Rancans

**Affiliations:** ^1^Department of Doctoral Studies, Riga Stradins University, Riga, Latvia; ^2^Department of Mental Health Care, Daugavpils Psychoneurological Hospital, Daugavpils, Latvia; ^3^Department of Psychiatry and Addiction Disorders, Riga Stradins University, Riga, Latvia; ^4^CBT Department, Latvian Association of Cognitive Behavioral Therapy, Riga, Latvia

**Keywords:** psychosis, schizophrenia, intervention, treatment, outcomes

## Abstract

**Background:** Patients with first-episode psychosis are mainly young people in the active phase of their social and professional lives, and psychosis is a serious disruption of normal life with high risk of disability. Integrated biopsychosocial early intervention treatment is crucial for patients with first-time psychosis episode. The purpose of this trial is to adapt the first early intervention program for patients with first-time non-affective psychosis in Latvia, and to investigate whether it is possible to integrate this kind of treatment approach in the frame of existing services and whether it provides better outcomes for patients than existing services.

**Design/Methods:** The study has a nonrandomized controlled design in a real-life environment. Participants are all consecutive patients presenting in the psychiatric emergency room with first-time non-affective schizophrenia spectrum psychosis episode (ICD criteria F23, F20) from a catchment area of 262,541 inhabitants, with urban and rural regions. The Latvian Early Intervention Program is a 6-month program developed from existing treatment guidelines and recommendations and adapted to a low-resource environment, integrated in an existing outpatient unit. This study aimed to test the hypothesis that the patients who received intervention have milder symptoms, higher functioning, and better adherence to outpatient treatment. The study primary aims are: 1) to establish and examine in practice the adapted early intervention for patients with first schizophrenia spectrum psychosis; 2) compare clinical and functional outcomes (including occupation, housing, and social relationships) between intervention treatment and standard treatment; and 3) compare the number of rehospitalizations, adherence to outpatient treatment, and assigned disability. Secondary aims are: 1) to compare full recovery status in both treatment groups at 12 months follow-up and 2) to develop recommendations for establishing early intervention programs in limited resource settings.

**Discussion/Conclusions:** Across the world, there is wide inequality in the availably and accessibility to early intervention treatment. This study will increase our knowledge in early intervention treatment approach and outcomes for patients with schizophrenia spectrum first psychosis episode in real-life working clinical practices. We hope to provide theoretical and practical aspects to develop strategies for early intervention service implementation in limited resource mental healthcare settings.

## Introduction

Over the past three decades, evidence has increased that early intervention is an effective treatment approach and secondary prevention for patients with a first psychosis episode (1) and its importance as a treatment for the early phase of schizophrenia (2). The first episode of psychosis most often starts in late adolescence or early adulthood, in the phase of life of active personal growth in education and social aspects. Psychotic disorders are strongly associated with suffering for young adults and their families (3) and predict poor long-term outcomes in lifetime perspective with professional and social deterioration (4) if not adequately treated.

The first 2–3 years after the manifestation of the first episode of psychosis have been considered as a critical period or window of opportunity (5) for contemporary schizophrenia treatment. In the 5 years after the first episode of psychosis, the relapse rates reach 70–80% in patients receiving regular care or standard treatment (6), which could cause a decline in personal, social, and professional functioning (7) and increase direct and indirect costs of mental healthcare (8). Psychotic disorders, including schizophrenia, are among the most common reasons for mental disorder caused disability in Europe (3, 9), with consequently increased economic load and lost workforce. Therefore, it is crucial to offer intense and adequate treatment such as early intervention for this group of patients.

The first early intervention services started almost 30 years ago (1), when psychiatric practices were introduced with comprehensive, intense, and young people-friendly treatment. Patients with psychotic disorders who received early intervention reached higher rates of remission, lower rates of residual positive and negative symptoms, lower rates of relapse, and had less substance abuse and better overall functioning (10–13). Moreover, specialized early intervention services for first-episode schizophrenia spectrum patients are shown to be effective to increase medication adherence and lower admission and rehospitalization rates (14), higher social and vocational activities (15, 16), and decreasing stress and discomfort for patients’ families (17).

The most highly functioning intervention teams are located in countries with developed mental healthcare systems (18). The most well-known early intervention services are in Australia (e.g., Early Psychosis Prevention and Intervention Centre (EEPIC), which is a part of the Orygen Youth Health Clinical Program) (19), the United Kingdom (e.g., LEO) (20), France (e.g., CJAA’D) (21), Denmark (OPUS) (22), the United States of America (23), Canada (13), and Scandinavian countries (TIPS) (24). A recent publication about European status in early intervention found that there were no significant differences between established intervention services and government expenditures in European countries, although countries with more psychiatrists and mental healthcare workers tended to have more established early intervention services (25). Nevertheless, there is lack of intervention services in Eastern Europe, mainly in the post-Soviet countries (26).

The authors were encouraged to adapt and start the first early intervention service in Latvia, which is a typical Eastern European country with one of the lowest levels of healthcare system funding in the European Union (EU) (27). A recent report of comparison of mental health of 30 European countries concluded that mental healthcare in Eastern Europe lacks integrated treatment; often, mental care is fragmented in separate hospital care and outpatient care and has an emphasis on institutions (28). In Latvia, there is no community treatment for patients with mental health disorders. In Latvia, the Ministry of Health is responsible for national health policy and the overall organization and functioning of the healthcare system—the state owns psychiatric hospitals, and Latvia currently has one of the highest rates of hospital beds in Europe (27), making psychiatric healthcare more hospital-based. The first initiative to shift from hospitals to outpatient care started in 2004 when Latvia joined the EU, and there was a dramatic decrease in the number of hospital beds. Almost 10 years later, the public health strategy was approved and had a strong influence on prevention and promoted intersectional approaches (29). The current health policy planning document, the Development Plan of Latvia for 2014–2020, continues these aims and highlights the importance of development on outpatient mental healthcare with planning long-term care for those with mental illness to provide not only medical care but also reintegration back into society (29). Psychiatric healthcare in Latvia is secondary care; if a patient has already been diagnosed with a psychiatric disorder, then their psychiatrist is a direct accessibility specialist. All psychiatric hospitals in Latvia are monoprofile and isolated from general hospitals. Four psychiatric hospitals in Latvia provide 24-h psychiatric emergency room services and are strictly organized by catchment area. Except for compulsory treatment or acute psychiatric states, including life threats to patients or others, all mental healthcare is voluntary based on patient preference and the patient is free to choose his or her outpatient psychiatrist or inpatient treatment facility. All psychiatric inpatient treatments and outpatient consultations with a psychiatrist (who has a contract with the government) are government-funded. Only in the capital Riga are outpatient mental healthcare centers separate from psychiatric hospitals; in the rest of Latvia, outpatient mental healthcare is administered by hospitals. Some centers offer patients a multi-professional team involvement; it is optional for patients and no programs for specific treatment have been used. Traditionally, in Latvia, the standard treatment for patients with psychosis or schizophrenia includes consultations with psychiatrists (mostly pharmacological treatment) based on the patients’ choices and subjective needs. In 2019, the Ministry of Health recognized the lack of integrated treatment in mental healthcare in Latvia and highlighted the problem and possible direction of action in the released strategy of improvement of mental healthcare accessibility for 2019–2020 (30). These facts indicate an opportunity for specialized treatment in practice, including case management and multidisciplinary teams for patients with first-time schizophrenia spectrum psychosis.

This study adapted the intervention program from existing guidelines and recommendations (31–34) without any funding. The program was implemented by reorganizing the existing resources in the outpatient center. To date, there are few “real-world” studies of regular mental healthcare systems with a sample of patients with first-episode psychosis.

The major objective of this study was to determine in routine Latvian outpatient settings whether the intervention treatment could be considered as more effective than standard treatment. To our knowledge, this is the first study on early intervention in the Baltic states; however, a single hospital initiative starting a separate psychosis ward (affective and non-affective) with a team of psychologists, psychiatrists, nurses, and modified case management after discharge in the Clinic of North Estonia Medical Centre Foundation (Tallinn, Estonia) has to be mentioned. We hope to fill the gap in theory and practice about the effectiveness and establishment of early intervention for patients with first-episode psychosis in limited resource settings.

## Hypothesis and Aims

The null hypothesis of this study is that patients who received intervention have milder clinical symptoms, higher functioning, increased employment, and better adherence to outpatient treatment. The study’s primary aims are: 1) to establish and examine in practice the adapted early intervention for patients with first schizophrenia spectrum psychosis; 2) to compare clinical and functional outcomes (including occupation, housing, and social relationships) between intervention treatment and standard treatment; and 3) to compare the number of rehospitalizations, treatment adherence, and assigned disability. The secondary aims are: 1) to compare full recovery status in both treatment groups at 12 months follow-up and 2) to develop recommendations for establishing early intervention programs in limited resource settings.

## Methods

### Design and Study Population

**Figure 1:**
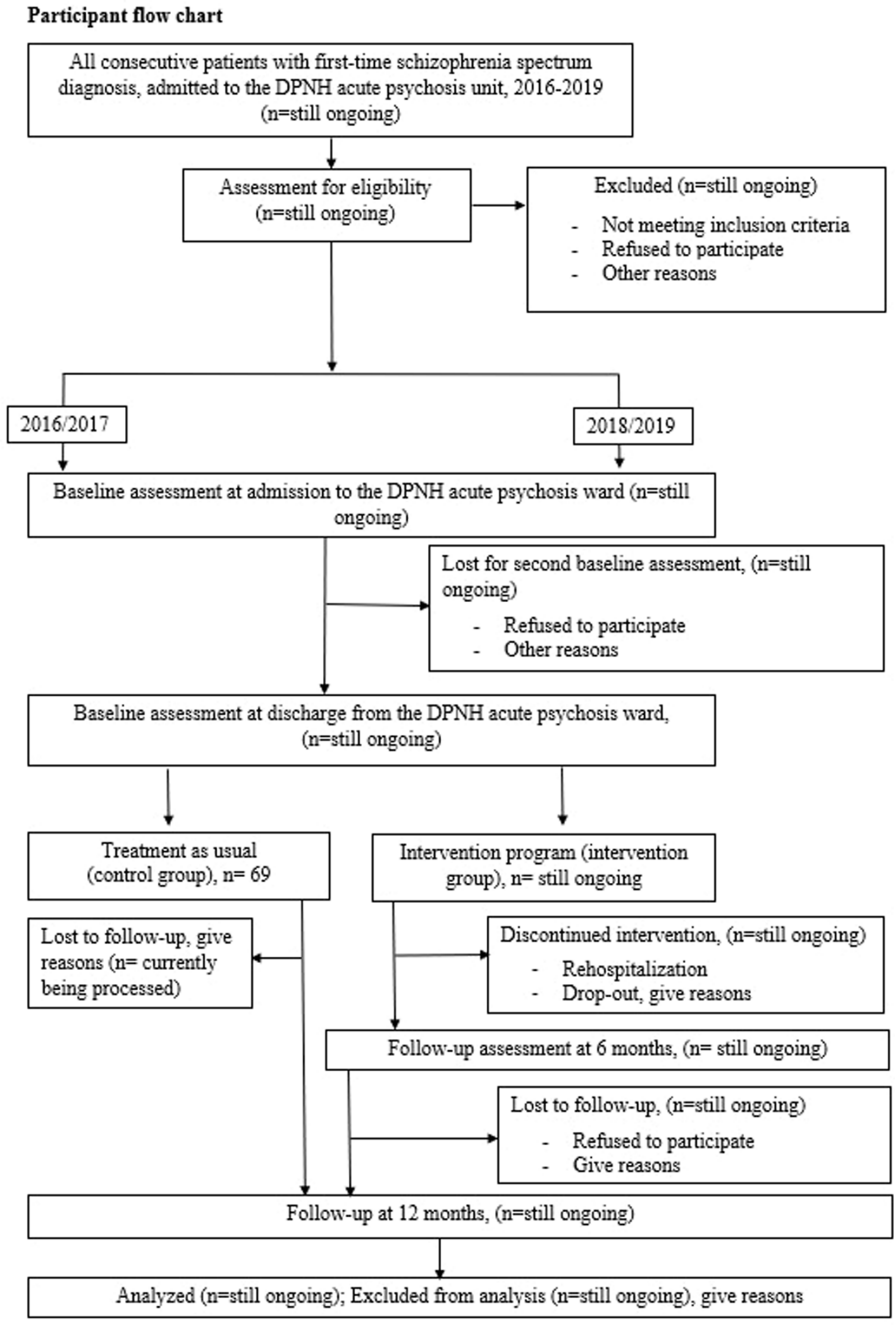
Participant flowchart.

This study aimed to investigate the effectiveness and applicability of a “real-life” working early intervention program. Therefore, the research was conducted in routine clinical practice, and the subjects were consecutive patients with the first episode of psychosis (FEP) from Daugavpils Psychoneurological Hospital (DPNH) during the recruitment period. This study was designed as a nonrandomized quasi-experimental controlled trial comparing early intervention treatment *versus* standard care. The study was conducted in the second biggest psychiatric hospital of Latvia—Daugavpils Psychoneurological Hospital. The hospital is specialized and exclusively offers psychiatric treatment with 24-h psychiatric emergency care. The Daugavpils Psychoneurological Hospital has a catchment area of approximately 262,541 inhabitants in 19 municipalities (two governmental-level cities and rural areas—this region is called Latgale) (35). As the psychiatric emergency care in Latvia, including treatment of psychotic disorders, is strictly based on catchment area basis, the study patient sample is representative for evaluating the incidence of psychotic disorders for the defined catchment area. The research authors met and offered all consecutive patients with first-episode psychosis admitted to DPNH during 2016–2019 the opportunity to participate in the study. All patients received treatment in a DPNH acute psychiatric ward according to guidelines (36). During their treatment in the hospital, all potential participants could meet with the study investigators and, after providing written and informed consent, were invited to participate in the study. The size of the control group is 69 participants, all consecutive patients with first-episode schizophrenia spectrum psychosis who met the inclusion criteria during 2016–2017. The control group patients agreed to participate in two basal assessments at admission and at hospital discharge and follow-up assessment after 12 months. The intervention group patients agreed to the same assessments and were invited to participate in a 6-month outpatient early intervention program with additional interviews before and after intervention treatment. The primary endpoint of intervention treatment was considered a second psychosis episode with rehospitalization. After receiving standard or intervention treatment, participants were naturally followed up for 12 months. The expected sample size of the intervention group is 30–40 consecutive patients with first-time schizophrenia spectrum psychosis from the defined catchment area. The work with the intervention group is still ongoing. **Figure 1** shows an overview of the patient flowchart.

### Eligibility Criteria

During the study period, the researchers examined the psychiatric emergency room register every 24 h for all first-time admissions to DPNH and identified from case records the patients diagnosed with non-affective schizophrenia spectrum psychosis by the emergency care psychiatrist. The study investigators then cross-checked the information with the treating psychiatrists and contacted patients in the DPNH inpatient units. Patients 18 and older were included if: 1) they had a diagnosis F20 or F23 as defined by the International Classification of Diseases, version 10 (37), by the research investigators (LB and IS); 2) this was the first psychosis episode in their lifetime; 3) they had read and signed an informed consent form; and 4) they were able and willing to participate in the study intervention treatment and assessment. The inclusion and exclusion criteria were highly nondiscriminatory when applied to consecutive patients with FEP. Therefore, we also included patients with substance abuse, but excluded them if they had an addiction specialist-approved drug addiction or the psychosis was clearly substance-induced. Other exclusion criteria were as follows: 1) organic etiology of the presenting psychotic symptoms, for example, brain/head injuries; 2) exogenous etiology of presenting psychotic symptoms; 3) comorbidity with intellectual disabilities (IQ < 70) based on previous medical history; and 4) use of antipsychotic medication for more than 4 weeks.

### Assessment Process and Instruments

All of the assessments in the study were applied by psychiatrists (LB and DK) with the supervision of a more experienced psychiatrist (IS). The kappa coefficient was used to evaluate the inter-rater reliability of the diagnosis and assessment procedure. The kappa values were interpreted as follows: excellent agreement (between 0.75 and 1.00), good agreement (between 0.60 and 0.74), fair agreement (between 0.40 and 0.59), and poor agreement (less than 0.40).

All of the participants were evaluated at the following time points: at the time of admission with FEP in a psychiatric ward (baseline = *T*_0_), at the time of discharge (*T*_1_), at follow-up assessment at 6 months (*T*_2_), and at follow-up assessment at 12 months (*T*_3_). In cases of withdrawal, one last assessment was performed at the final consultation (*T* *_x_*) (**Table 1**).

**Table 1:** Assessment tools used at each evaluation time point.

	*T*_0_	*T*_1_	*T*_2_	*T*_3_	*T*_x_
	Admission to acute psychosis unit	Discharge from acute psychosis unit	Follow-up at 6 months	Follow-up at 12 months	Before withdrawing
Socio-demographic data	x		x	x	x
Help-seeking behavior schedule	x				
NOS-DUP	x				
SAPS	x	x	x	x	x
SANS	x	x	x	x	x
CDSS	x	x	x	x	x
SAI-E	x	x	x	x	x
GAF	x	x	x	x	x

NOS-DUP, Nottingham Onset Schedule-Duration of Untreated Psychosis Version; SAPS, Scale for the Assessment of Positive Symptoms; SANS, Scale for the Assessment of Negative Symptoms; CDSS, Calgary Depression Scale for Schizophrenia; SAI-E, Schedule for the Assessment of Insight—Extended Version; GAF Global Assessment of Functioning.

All of the data were collected during face-to-face semi-structured clinical interviews and the expected time to complete all of the questionnaires and instruments was up to 60 min. In the baseline assessment, a semi-structured clinical interview contained three blocks: 1) sociodemographic data, 2) help-seeking behavior, and 3) clinical assessment with instruments. Additional information was derived from medical files, including clinical records from the attending psychiatrist, and used for cross-checking the information given by the patient. The provided information was checked by the closest family members or the patients’ significant others.

#### Socio-Demographic Data

At the baseline, the following socio-demographic information were collected: age, sex, education level, relationship status (including being a parent), living arrangements (independently, with family, an/or with relatives), vocational status (employment: yes/no; studies: yes/no, additionally, time of employment or unemployment; the type of studies: full-time/part-time), family history of psychiatric disorders, comorbidities, and suicide attempts during lifetime.

#### Help-Seeking Behavior

To explore help-seeking behavior during the interview, we used a study author’s conducted questionnaire that contained the following information: 1) help-seeking initiator (patient himself/herself, family members, friends/colleagues, medical workers, or other); 2) first contact with healthcare practitioners before admission to the psychiatric emergency room (general practitioner, psychiatrist, other, or none); and 3) how the patient was brought to the psychiatric emergency room (by himself/herself, by family members, by ambulance, or by ambulance with police escort).

#### Clinical Assessment With Instruments

To assess the different aspects of the patients’ psychopathology, we used the following schedules and scales:

- The duration of untreated illness and the duration of untreated psychosis were evaluated using the Nottingham Onset Schedule-Duration of Untreated Psychosis version (NOS-DUP). The NOS-DUP is a short guided interview for recording several time points in the onset of psychosis. The NOS-DUP interview is designed to be administered by a clinician at the baseline evaluation. Onset is defined as the period between the first reported/observed changes in mental state/behavior to the development of psychotic symptoms (transition into psychosis). The NOS-DUP outlines several ways that the DUP can be defined. This study used the following definitions: 1) duration of untreated psychosis: duration from the first obvious psychotic symptoms to the start of anti-psychotic treatment; and 2) duration of untreated illness: from the prodrome initiation to the start of anti-psychotic treatment. To conduct the NOS-DUP interview, the following structured and standardized steps were taken: the NOS-DUP was administered as near to the time of illness onset as possible after other schedules (covering history and mental state) have been administered, and the interview includes open-ended questions and standardized checklists (38). Similar to other studies, the admission day to the psychiatric hospital was considered the starting point for adequate treatment (39).
- The positive symptoms of the schizophrenia spectrum psychotic disorder were assessed with the Scale of the Assessment of Positive Symptoms (SAPS) (40) and the negative symptoms of the schizophrenia spectrum psychotic disorder were assessed with the Scale of the Assessment of Negative Symptoms (SANS) (41). The SAPS is a widely used assessment scale that includes four main domains of psychotic positive symptoms (schizophrenia spectrum): hallucinations, delusions, bizarre behavior, and positive formal thought disorder. The SANS is a widely used assessment scale that includes five main domains of psychotic negative symptoms (schizophrenia spectrum): affective flattening or blunting, alogia, avolition–apathy, anhedonia–asociality, and disturbed attention. Symptoms included in the SAPS and SANS were evaluated with a score from 0 (none) to 5 (severe). For the SAPS and SANS, we used the total score across all domains for a better comparison between these variables at admission and discharge (42).
- The Global Assessment of Functioning (GAF) was used to evaluate functioning (43). The scale has demonstrated good inter-rater reliability in those with psychosis (44). The GAF is a widely used scale measuring functioning during the previous month. It has 10 sections that provide a description of overall functioning based on the interviewer’s opinion regarding the level of general activity and functioning of patients (severity). The evaluation step has 10 scores. The lower score of 1–10 is applied if there is a persistent inability to maintain even minimal personal hygiene and the person is in danger of severely hurting himself/herself or others, and the higher score of 91–100 is applied if there are no symptoms and the person has superior functioning.
- The Calgary Depression Scale for Schizophrenia (CDSS) scale was used for depressive symptom evaluation (45). The scale is designed to assess the symptoms of depression in patients with schizophrenia. The scale contains nine questions (scored from 0, absent, to 3, severe) asked by the interviewer. The scale has demonstrated good reliability and validity with a specificity of 77% and a sensitivity of 92% to diagnose depressive episodes starting with a score of 6 (45).
- The Scale for the Assessment of Insight (SAI) (46) was used to evaluate the patient’s insight about their disorder, adherence to treatment, and medication use. It is a well-established measurement of insight in patients with psychiatric disorders and contains nine items: six items are scored 0–2 and three items are scored 0–4, for a scale rating range of 0–24 (higher scores represent good insight). The expanded version (SAI-E) contains three sub-items the interviewer uses to evaluate the patients’ compliance with treatment based on one to seven previously asked questions (47).

#### Clinical Record Data

During the baseline and follow-up assessments, all available information was used from DPNH registers, medical records, and treating psychiatrists case notes to collect the following information: days spent in the hospital, emergency visits, outpatient visit contacts, number of readmissions (voluntary and involuntary), suicide attempts and suicide, disability status, and psychopharmacological treatment administered. Pharmacological treatment in the study sample is coded according to the Anatomical Therapeutic Chemical (ATC) classification index. This study evaluates only antipsychotic medication (ACT code N05A, excluding lithium) used in psychosis treatment, as this is the first-line treatment in most psychosis guidelines in Europe (32) and Latvia (36).

### Outcome Measures

#### Primary Outcomes

The primary outcomes were assessed at baseline and at the 12-month follow-up comparing both treatment groups using the following instruments: 1) Clinical psychopathology was assessed using SAPS, SANS, CDSS, and SAI-E. The functional outcome was measured using GAF and vocational status (working or studying). The main measure was the difference between the total scores in both groups when comparing the baseline assessment and 12-month follow-up. Social functioning was explored in different social roles (interpersonal relationships, establishment of close personal relationships with a partner, and independent living from relatives) based on patients’ answers during the clinical interview. 2) The number of rehospitalizations, days spent in hospital, and current disability status was counted using the DPNH register and all available medical records about the last 12-month period. Treatment adherence was measured by number of psychiatrist outpatient appointments during the last 12 months.

#### Secondary Outcomes

Secondary outcomes include full recovery status after FEP at 12 months follow-up in both groups. Full recovery is defined as a stable remission of both negative and positive symptoms (evaluated as 2 points or less in the SAPS and SANS global items), currently engaged in work or education, a GAF score over 60, and no psychiatric admissions to the hospital or disability during the last 12 months (48, 49). The recommendations of applicability of the early intervention program will be based on obtained study results and practical observations during the implementing process of Latvian Early Intervention Program (LAT-EIP) in real-life working everyday psychiatric practice.

### Safety Assessments

Intervention is not expected to cause any adverse effects. The safety assessment will focus on adverse effects of psychopharmacological treatment and well-known adverse effects in patients with psychotic disorders: 1) suicide attempt or suicide, 2) substance abuse, and 3) violence against self or others. In the assessment points, the researchers monitored the possible adverse incidents and immediately contacted the treating psychiatrists when any clinical worsening and suicidal risk were identified. In cases of patient death, the cause was attained from patient medical records.

### Latvian Early Intervention Program

#### Theoretical Background of Intervention

This is the first time an early intervention program for patients with schizophrenia spectrum psychosis has been developed in Latvia (LAT-EIP). The study authors constructed the LAT-EIP in an *ad hoc* manner using the experience-based approaches described in research literature (14, 22, 23, 50, 51), well-known guidelines (31, 32, 52) and manuals and handbooks to adapt the program for Latvia’s mental healthcare system. In the LAT-EIP development process, the study authors organized focus groups with expert researchers in psychiatry and psychology, clinicians working with FEP patients, and the administration of the hospital in order to identify the most relevant interventions to be included in the program The LAT-EIP structure and intervention elements are based on the Australian Clinical Guidelines for early psychosis (31). Strategies for managing organizational and financial aspects were found in the “Implementing Early Intervention in Psychosis: A Guide to Establishing Early Psychosis Services” by Jane Edwards and Patrick McGorry, 2006 (53). After developing a structure of the LAT-EIP with a description of the elements and piloting the LAT-EIP in a small sample of patients with FEP, the study authors conducted a real-life working version of the LAT-EIP protocol (**Table 2**), which was confirmed by local authorities (administration of DPNH) and the Ethics Committee of Riga Stradins University (no. 114/21.12.2017). The LAT-EIP consists of six main elements: 1) case management, 2) psychiatrist appointments, 3) family and individual psychoeducation, 4) techniques based on third-wave cognitive behavioral therapy (CBT) and social skills training, 5) consultations with vocational specialist, and 6) a nurse who will help with medication use and monitor the side effects. The LAT-EIP length is 6 months, which the authors consider the optimal time period, considering the patients’ needs and providing multidisciplinary teamwork in limited resource settings. The LAT-EIP is developed to be easily tailored for individual patient needs. The study participants are encouraged to engage in all of the LAT-EIP interventions by the treating psychiatrist; nevertheless, the priority is the patient’s opinion, and their voluntarily chosen activities are strongly respected.

**Table 2:** Structure of the LAT-EIP for patients with first-episode psychosis.

Intervention^a^ (months)	Psychiatrist	Psychoeducation with family	Psychologist	Vocational specialist
1	1x/7 days1x/14 days	First session		
2	1x/14 days1x/21 days		2x/28 days	First consultation
3	1x/21 days1x/21 days	Second session	2x/28 days	Second consultation
4	1x/28 days	Third session	2x/28 days	Meeting at SEA
5	1x/28 days			
6	1x/28 days	Booster session (optional)	Booster session (optional)	

LAT-EIP, Latvian Early Intervention Program; SEA, State Employment Management.

^a^All of the intervention times and places are tailored to the patient needs and specialist availability and coordinated by the case manager.

#### Case Management

The case manager is involved in the patient care for 6 months to provide the coordination of the LAT-EIP realization in practice. The intervention plans are discussed between the patient, psychiatrist, and case manager to create the most acceptable plan for patients to visit the psychiatrist, psychologist, or vocational specialist. The case load is approximately 10 patients, and patients can reach their case manager every day from 0800 to 2000 via mobile phone (including weekends and holidays); if a message is sent after hours, the case manager will call back the next day. The contact with the case manager is provided via phone calls, messages, and face-to-face visits. The case manager’s other tasks include establishing confidential and supportive contact with the patient and at least one family member or significant other, sending reminders about specialist appointments or rescheduling the visit if necessary, monitoring the patient’s well-being and ensuring the availability of a psychiatrist within the next 48 h in case of sudden worsening of mental health status, and obtaining feedback about medication use and treatment compliance. A crisis plan was developed for each patient in which the first step is to contact the case manager or, if this is not possible, to provide help in a 24-h working psychiatric emergency room.

#### Psychiatrist Appointment

The psychiatrist is offering psychopharmacological treatment according to Latvian guidelines on schizophrenia treatment and management, including the management of a first-time psychosis episode (36). During team meetings, the psychiatrist plays a leading role by providing the best LAT-EIP option plan so that the patient can achieve his or her individual treatment goals.

#### Family Psychoeducation

To ensure better engagement with the treatment program, as one of the primary actions, the study authors emphasized the early involvement of family members. In this study, psychoeducation was applied as uni-family intervention. The definition we used is from the NICE guidelines in which psychoeducation is generally defined as information provided about a condition and its management (32). After the first contact with the patient, the psychoeducational family intervention was offered, and the team members always tried to contact at least one family member or emotionally close person and motivate them to participate in a psychoeducational session. This approach was primarily built on friendly, emphatic, optimistic, and open communication and collaboration.

Family psychoeducation included three 45-min sessions, and two booster sessions could be offered, if needed. The sessions were developed to help the family identify the psychotic disorder course, management, and prognosis and plan strategies for coping with future difficulties. Family psychoeducation was followed by two family psychosocial intervention manuals, Compton and Broussard’s “The First Episode of Psychosis: A Guide for Patients and Their Families” (54) and Kuipers, Leff, and Lam’s “Family Work with Schizophrenia: A Practical Guide” (55). Uni-family (including significant others by patient invitation) sessions are led by the treating psychiatrist with the case manager. The time and date of the sessions can be adapted to the family’s needs and mental health professionals’ availability.

The psychoeducation consists of three informative sessions (the booster session is optional) that cover the following: 1) information on the main clinical and epidemiological features of psychosis, including long-term outcomes, risk to children and impact on professional preferences, importance of adherence to therapy, pharmacological and non-pharmacological treatment options are discussed, and the possible side effects and benefits of treatment; 2) training on early warning signs, how to recognize them, and the importance of maintenance therapy as a prevention strategy for relapse (second psychosis), including non-pharmacological evidence-based psychosocial interventions; for each family, the crisis plan was discussed for psychiatric emergency situations, including substance abuse and suicidal behavior, and the social skills training with a main focus on strengthening problem-solving skills; and 3) the importance of the family’s role in adherence to treatment and toward functional recovery and reintegration into employment and/or educational activities, the role of the family as emotional support during the development of social, professional, affective, and romantic relationships, taking into account that promoting patient independence is one of the main goals of treatment. In all of the sessions, the families are warmly encouraged to ask questions, discuss individual needs, and provide feedback at the end of the session. All of the details about the family education can be obtained from the authors.

#### Elements of Cognitive Behavioral Therapy and Social Skills Training

Structured CBT is not used in this study. Instead, the patients are offered need-based psychological interventions with elements of CBT in individual sessions with a certified clinical psychologist. The authors developed six sessions with one booster session for the psychological intervention based on third-wave CBT, including elements of acceptance, commitment therapy, and mindfulness (56). Additionally, specific individual social skills training was emphasized through CBT sessions. All of the sessions use a clear session structure and repetition and brief mindfulness exercises (57). There are possible adaptions for each patient; the individual approach is discussed during team meetings between the psychologist, psychiatrist, and patient. Each session length is approximately 30–40 min, and the frequency is tailored to the patient attending sessions twice or once per month. The psychological intervention focus is on personal value-based actions, acceptance of the psychotic experience, and developing non-judgmental attitudes toward the experience and the patient himself or herself (58). The important part of the work in the psychological intervention is anxiety, post-psychotic emotional symptom reduction, and the development of coping mechanisms, as well as social skills training (for example, developing problem-solving techniques) considering the individual patient needs. The psychological intervention is not supposed to be administered and evaluated separately, but is a part of the program and promotes adherence with the treatment plan, psychopharmacological treatment, and other specialists. **Table 3** shows the structure of the six sessions.

**Table 3:** Overview of the adapted individual sessions with the clinical psychologists.

Session 1	Introduction of structure and aims of psychological intervention Establishment of honest and open therapeutic relationships Introducing values-based actions Mindful of body and breath exercises
Session 2	Warm-up exercise Introduction with the concept of acceptance of psychotic experience Acknowledging the individual emotional difficulties and development of coping mechanisms Mindfulness breathing exercise Out-of-session planning activity exercise: value-based action plus mindfulness practice
Session 3	Warm-up exercise Mindfulness of body and stretching exercise Review of out-of-session exercise Identification of individual obstacles in value-based actions and developing coping strategies Social skills training practice Mindfulness breathing exercise Out-of-session planning activity exercise: value-based action plus mindfulness practice
Session 4	>Warm-up exercise Mindfulness eating exercise Review of out-of-session exercise Introduction to short vignettes that are comparable to patient's experiences Strengthening the acceptance and commitment of personal experiences and values Review of social skills training practice, introduction of alternative solutions Mindfulness breathing exercise Out-of-session planning activity exercise: value-based action plus mindfulness practice
Session 5	Warm-up exercise Mindfulness walking exercise Review of out-of-session exercise Review of learning and progress Acknowledging individual strengths and weaknesses; developing strategies to overcome them Mindfulness breathing exercise Out-of-session planning activity exercise: value-based action plus mindfulness practice
Session 6	Warm-up exercise Mindfulness walking exercise Review of out-of-session exercise Review of learning and progress Setting goals for value-based future actions Refreshing coping mechanisms and social skills Mindfulness breathing exercise Wrapping-up exercise
Booster session	Warm-up exercise Review of learning and progress Strengthening the coping mechanisms Refreshing social skills exercises Revising different mindfulness exercises Wrapping-up exercise

#### Consultations With Vocational Specialists

All of the participants who reach symptomatic remission are offered two consultations with vocational specialists in outpatient settings. The collaboration contract was signed between DPNH and the State Employment Agency (SEA). A vocational specialist ensured two consultations and a third scheduled meeting in the SEA office. During the first consultation, the client was asked about his or her preferences and his or her education and previous work experiences summarized, and consequently a client profile is created, including guided resume writing. In the second consultation, the possible options are offered in educational activities organized by SAE or job openings found by the vocational specialist. The client was encouraged to seek opportunities by himself/herself and ask for support and advice when it is needed for filling out applications or interviews. During the second interview, practical tasks were offered, such as job interview role-playing and self-presentation techniques. The third consultation in the SAE office included the contract and placement in educational courses offered by SAE and completely cost-free with cash benefits for attending the course and finishing it (provided by SAE). The vocational specialist was invited to the LAT-EIP team meeting at least once in 2 months, and after all of the consultations with clients (two to three individual consultations twice a month), the vocational specialist had a brief team meeting with the treating psychiatrist and case manager.

#### Withdrawal From Program

LAT-EIP philosophy is the early intervention program is patient-driven and tailored; therefore, the patient has the right during the program to refuse specific elements or interventions and continue to work with other interventions. If the patient was reluctant to be treated at all, the team remained in contact with the patient and the family to find a common focus for collaboration to motivate the patient to continue treatment. In this study, a dropout is defined as a situation when the patient stopped being reachable via mobile phone (quit answering phone calls and did not respond to messages), resulting in three missed appointments. If dropping out occurred, the team members tried to involve the patient and his or her family in one last meeting with the psychiatrist and case manager to ascertain the reasons for dropping out and to conduct a final assessment for the possible outcome evaluation. In the initial phase of the program, the participants were informed that they could refuse further treatment at any time during the program and they have the right to forbid the use of their medical records. To continue follow-up work after patient withdrawal, at the beginning of the study, the importance of using the data from medical records to obtain information for possible outcome measures was explained to the patients. A request to use patient data after withdrawal was included in the informed consent.

## Statistical Analysis

The data were analyzed using IBM SPSS v.25. The Kolmogorov–Smirnov test was used to evaluate the normal distribution of continuous data. For normally distributed variables, the means (*M*) and standard deviation (SD) were applied for nonparametric numeric data: medians (Md) and interquartile ranges (IQR). Demographics and baseline characteristics were summarized and assessed for comparability between the intervention and control groups. Adjustment for baseline measures was used as this increased the statistical power and accounted for the regression to the mean (59). All of the continuous outcomes were analyzed as changes from the baseline with random intercepts for the participants and adjusted for the baseline measure. The study hypothesis was tested on the main effect for the group. For comparison between categorical variables, we used Pearson’s chi-squared test or Fisher’s exact test, with phi for the estimation of the effect size (weak effect: phi < 0.3, medium: 0.3–0.5, and large: >0.5). The paired samples *t* test was used to determine whether improvement over time occurred within both groups. To identify predictors and moderators related to the standard or intervention treatment outcomes, mixed regression methods were used. Univariate and multivariate logistic regression was used to find the association between the variables. The results were expressed in odds ratios (ORs) with a 95% confidence interval (CI). The Kaplan–Meier method was used to estimate the time to remission (in months) and remission rates. The log-rank test was used for statistical comparison between the two patient groups. The Cox proportional hazards regression was used to calculate the hazard ratios (HRs) to evaluate the influence of covariates for the adjusted analyses. The secondary outcomes were analyzed using relevant tests: the Mann–Whitney test, *U* tests, and the Kruskal–Wallis test with *r* as the effect size. We also explored Cohen’s *d* and the effect size for the changes in positive and negative symptomology at the time of admission and discharge and compared between the groups. To describe the relationships, we used Spearman’s two-tailed correlation coefficient analysis (*r*_s_). In cases of nonexistent outcome measures (due to withdrawal, dropping out, or being lost to follow-up), the pattern of missing data and the assumption of missing data at random (MAR) was explored. The level of significance for all of the statistical analyses tasks was set at 0.05.

### Ethics Statement

This study was approved by the Ethics Committee of Riga Stradins University, Riga, Latvia (no. 114/21.12.2017). Before signing informed consent, all of the patients were provided with written and verbal information on the study and given unlimited time to ask questions. All of the participants were informed both verbally and in writing that they could withdraw from the study at any time without any consequences for their further treatment. The authors ensured that all of the specialists involved in this study were properly qualified in their professional field and fully informed about their study-related duties during the research process. This study was conducted in accordance with globally accepted standards of good clinical practice and in agreement with the Declaration of Helsinki and with national and local regulations.

## Discussion

This study investigated and practically examined the implementation of a first early psychosis program in an environment without community care by reorganization of existing resources. For many young people with psychosis, the introduction to mental services could be considered traumatic (14). Often, the first contact with a mental healthcare specialist is in a psychiatric emergency room and may include first-time admission in a psychiatric hospital in wards with disabled and chronic patients. The system in Latvia does not provide community care, assertive care, or individual case management for patients with psychiatric disorders, and it is left to the patient to voluntarily visit a psychiatrist by choice. It is crucial to use innovative approaches to assist stakeholders and policy makers in the development and spread of youth- and family-friendly early intervention services to ensure an optimistic first contact with services and minimize adverse effects such as unemployment, loss of life opportunities, and physical health comorbidities (60).

The literature documents disagreements on the type of early intervention model that is most appropriate for patients and applicable and feasible in real-world mental healthcare structures (61) and debates on obstacles when early intervention centers are established within the public Department of Mental Healthcare (62). In Latvia, healthcare expenditures are among the lowest compared to other countries; nevertheless, the situation in similar to other Eastern Europe countries with similar policy makers’ attitudes toward mental healthcare (28). The goal of this study was to develop a working program for the early intervention team for routine outpatient mental healthcare practice without additional funding. During the research process, the authors not only explored the background and applicability of reorganizing existing structures and staff but also compared the two groups of patients with statistical approaches regarding which treatment option was better and more effective. There is evidence in the literature of the early intervention’s superiority over standard treatment (63), but most studies are conducted in separate, funded institutions and projects that are almost impossible to replicate in real-world settings. The authors chose a model of early intervention team integration in a government-funded mental hospital, acknowledging that integration in already existing institutions has its advantages and disadvantages (64); nevertheless, it is one of the most realistic ways of implementing a new service in limited resource settings. When the authors started this study in Latvia, there were multidisciplinary teams working with patients with psychiatric disorders only in Riga, but there were no programs for specialized patient groups. There is an opinion in the literature that patients with psychosis are in a patient group with specific needs (14, 18) based on the fact that the LAT-EIP is the first specialized program for patients with psychosis in Latvia.

Although the LAT-EIP was developed following the best practice advice and experience from other countries, our program emphasizes the need for individualized approaches to patients, creative solutions for obtaining resources, and flexible administration and team members. Our core team specialists are a psychiatrist, a case manager, a psychologist, a nurse, and a vocational specialist, as previously described (14, 51, 64). The intervention program included empirical and scientific methods (65), psychiatrist appointments, case management, CBT-oriented psychological interventions, and vocational specialist consultations. As unemployment is a major problem among patients with psychotic disorders and is associated with poor social and economic inclusion as well as poorer life functioning (66), this study highlighted the need for the early involvement of vocational specialists via collaboration with the State Employment Agency.

To compare both treatment options, the standard treatment and intervention treatment, we selected assessment tools on the basis of their wide use in research trials (12, 51, 64) and therefore were able not only to evaluate the outcomes in our patient sample but also compare it to other studies. We aim to implement the early intervention model, which would be applicable to other limited resource settings in this region. At the end of this study, we plan to demonstrate the relevant statistics with the evaluation of the two treatment groups, the standard and intervention treatment, to confirm the hypothesis that the intervention is more effective than the standard treatment and could be implemented in the existing mental healthcare system. Practical observations will be included in the development of recommendations for mental healthcare specialists and policy makers to suggest the process of early intervention implementation in the available structures and institutions of the mental healthcare system in Latvia.

## Limitations and Strengths

The strength of this study is that we were able to include a representative number of non-affective patients with first-time psychosis presenting to a secondary mental healthcare service in the defined region, which combines rural and urban areas. Nevertheless, the major strength of this study is the real-life approach that the LAT-EIP is being established in already existing organizational structures and it is possible to evaluate the intervention *versus* standard treatment effectiveness in a real-world patient sample. As this study was conducted in the second largest psychiatric hospital in Latvia and all five national psychiatric hospitals have similar administrative structures, the authors are confident that the LAT-EIP protocol could be considered as a pilot study representing the basis for implementation in any of the psychiatric hospitals in Latvia and possibly other Eastern European regions.

This study had several limitations that must be acknowledged. The first limitation is the study design; it was a nonrandomized study, but a quasi-experimental study with the control group of all consecutive patients with first-episode psychosis were recruited before the intervention group. No reforms to the mental health system were conducted during the recruitment and intervention periods on the national level regarding psychiatric treatment options. However, the authors emphasize that this study has a real-life approach which allows making conclusions not only about patients but also on an administrative mental healthcare system level. The second limitation is that the sample size is one of five regions in Latvia and the incidence of patients with first-time psychosis in this region is comparable with the literature; the sample size could be considered small and could have an impact on the statistic power. It has to be taken into account that in the study were recruited all consecutive patients from one definite catchment area; the sample size covers the second biggest region of Latvia and is representative. Third, even though standard treatment and intervention treatment were ensured by different specialists, the evaluation was conducted by the same assessors, which may cause performance bias. In contrast, the effectiveness of the intervention could be biased by the short duration of the intervention treatment in this study; other studies have demonstrated the importance of extended interventions, confirming better effectiveness with prolonged early intervention in psychosis (67). Lastly, in this study, we did not include any cognitive testing which could affect clinical and functional outcomes. The authors strongly recommend adding these measurements when the early intervention will be expanding in the defined area and the protocol will be replicated. Therefore, it is necessary to implement early intervention programs in other mental health services in Latvia for a more valid and randomized methodology.

## Conclusions and Future Implementation

The LAT-EIP study fills the gap in the current knowledge about implementing early intervention for patients with psychosis in specific non-community care mental healthcare systems with limited resource settings. By investigating the clinical and functional outcomes and effectiveness of the practical intervention protocol, we hope to develop recommendations for implementing feasible early interventions in Latvia for mental healthcare professionals, treatment providers, and policy makers. Implementing this study initiative in every psychiatric practice in Latvia should be a major benefit to individuals with first-time psychosis, their families, and society in general.

## Data Availability Statement

The datasets analyzed in this manuscript are not publicly available. Requests to access the datasets should be directed to lieneberze@gmail.com.

## Ethics Statement

The studies involving human participants were reviewed and approved by Ethics Committee of Rigas Stradins University. The patients/participants provided their written informed consent to participate in this study.

## Author Contributions

ER and LB contributed to the study conception and design. LB, IK, IS, JL, and ER were involved in the development of the intervention program and created the study protocol. SK, IS, and JL were involved in the study organization, logistics, and implementation of the intervention program in outpatient settings. SC, LB, and DK were involved in the patient recruitment and data management. IS and JL are experts in psychiatry and IK is an expert in psychological interventions. LB coordinated the study and statistical counseling. LB wrote the first draft of the manuscript. ER, SC, IK, SK, IS, and JL provided critical revisions of the manuscript. All of the authors contributed to the manuscript revision and read and approved the final version.

## Funding

This study did not receive any funding. The LAT-EIP team was organized in the DPNH outpatient center and included in the employees’ working hours.

## Conflict of Interest

The authors declare that this study was conducted in the absence of any commercial or financial relationships that could be construed as potential conflicts of interest.
